# Supplementary material for: Sex- and strain-dependent effects of ageing on sleep and activity patterns in Drosophila
Source: PLoS One. 2024 Aug 16;19(8):e0308652. doi: 10.1371/journal.pone.0308652 (PMC11329114; doi:10.1371/journal.pone.0308652)
Supplement: S1 Fig — (PDF) [file pone.0308652.s001.pdf]

S1 Fig. 2-way ANOVA results for all figures.

Figure	Panel	Strain	Phenotype	Source of variation	% of total variation	P value	P value summary
2	G	CS	Day Activity	Interaction	3.243	0.0017	**
2	G	CS	Day Activity	Age	27.79	<0.0001	****
2	G	CS	Day Activity	Sex	22.17	<0.0001	****
2	H	Dah	Day Activity	Interaction	6.128	0.0024	**
2	H	Dah	Day Activity	Age	1.886	0.2101	ns
2	H	Dah	Day Activity	Sex	1.571	0.0525	ns
2	I	w1118	Day Activity	Interaction	11.55	<0.0001	****
2	I	w1118	Day Activity	Age	5.904	0.0009	***
2	I	w1118	Day Activity	Sex	0.3959	0.2858	ns
2	J	CS	Night Activity	Interaction	1.689	0.0904	ns
2	J	CS	Night Activity	Age	38.86	<0.0001	****
2	J	CS	Night Activity	Sex	1.065	0.0431	*
2	K	Dah	Night Activity	Interaction	3.814	0.0088	**
2	K	Dah	Night Activity	Age	11.24	<0.0001	****
2	K	Dah	Night Activity	Sex	11.9	<0.0001	****
2	L	w1118	Night Activity	Interaction	5.143	0.0049	**
2	L	w1118	Night Activity	Age	1.902	0.1839	ns
2	L	w1118	Night Activity	Sex	0.1616	0.5202	ns
3	B	CS	Morning Anticipation	Interaction	0.2047	0.7655	ns
3	B	CS	Morning Anticipation	Age	42.9	<0.0001	****
3	B	CS	Morning Anticipation	Sex	14.95	<0.0001	****
3	C	Dah	Morning Anticipation	Interaction	3.15	0.002	**
3	C	Dah	Morning Anticipation	Age	18.99	<0.0001	****
3	C	Dah	Morning Anticipation	Sex	30.17	<0.0001	****
3	D	w1118	Morning Anticipation	Interaction	2.599	0.0048	**
3	D	w1118	Morning Anticipation	Age	11.65	<0.0001	****
3	D	w1118	Morning Anticipation	Sex	38.57	<0.0001	****
3	E	CS	Evening Anticipation	Interaction	1.438	0.053	ns
3	E	CS	Evening Anticipation	Age	33.34	<0.0001	****
3	E	CS	Evening Anticipation	Sex	20.13	<0.0001	****
3	F	Dah	Evening Anticipation	Interaction	0.5214	0.5723	ns
3	F	Dah	Evening Anticipation	Age	6.427	<0.0001	****
3	F	Dah	Evening Anticipation	Sex	33.13	<0.0001	****
3	G	w1118	Evening Anticipation	Interaction	11.01	<0.0001	****
3	G	w1118	Evening Anticipation	Age	21.89	<0.0001	****
3	G	w1118	Evening Anticipation	Sex	29.38	<0.0001	****
4	G	CS	Day Sleep	Interaction	2.492	0.0011	**
4	G	CS	Day Sleep	Age	25.21	<0.0001	****
4	G	CS	Day Sleep	Sex	33.18	<0.0001	****
4	H	Dah	Day Sleep	Interaction	6.464	0.0006	***
4	H	Dah	Day Sleep	Age	5.927	0.0012	**
4	H	Dah	Day Sleep	Sex	10.33	<0.0001	****
4	I	w1118	Day Sleep	Interaction	4.426	0.0005	***
4	I	w1118	Day Sleep	Age	9.719	<0.0001	****
4	I	w1118	Day Sleep	Sex	27.64	<0.0001	****

4	J	CS	Night Sleep	Interaction	3.658	0.0003	***
4	J	CS	Night Sleep	Age	50.59	<0.0001	****
4	J	CS	Night Sleep	Sex	3.771	<0.0001	****
4	K	Dah	Night Sleep	Interaction	8.279	<0.0001	****
4	K	Dah	Night Sleep	Age	11.73	<0.0001	****
4	K	Dah	Night Sleep	Sex	10.06	<0.0001	****
4	L	w1118	Night Sleep	Interaction	2.736	0.0334	*
4	L	w1118	Night Sleep	Age	3.353	0.0139	*
4	L	w1118	Night Sleep	Sex	20.47	<0.0001	****
5	A	CS	Day Sleep Bout Number	Interaction	3.018	0.0003	***
5	A	CS	Day Sleep Bout Number	Age	24.66	<0.0001	****
5	A	CS	Day Sleep Bout Number	Sex	33.26	<0.0001	****
5	B	Dah	Day Sleep Bout Number	Interaction	11.04	<0.0001	****
5	B	Dah	Day Sleep Bout Number	Age	7.128	<0.0001	****
5	B	Dah	Day Sleep Bout Number	Sex	13.4	<0.0001	****
5	C	w1118	Day Sleep Bout Number	Interaction	3.019	0.0055	**
5	C	w1118	Day Sleep Bout Number	Age	13.78	<0.0001	****
5	C	w1118	Day Sleep Bout Number	Sex	27.38	<0.0001	****
5	D	CS	Night Sleep Bout Number	Interaction	2.994	0.0129	*
5	D	CS	Night Sleep Bout Number	Age	35.52	<0.0001	****
5	D	CS	Night Sleep Bout Number	Sex	0.4701	0.1893	ns
5	E	Dah	Night Sleep Bout Number	Interaction	4.237	0.0152	*
5	E	Dah	Night Sleep Bout Number	Age	8.532	0.0001	***
5	E	Dah	Night Sleep Bout Number	Sex	0.5814	0.2278	ns
5	F	w1118	Night Sleep Bout Number	Interaction	4.392	0.0029	**
5	F	w1118	Night Sleep Bout Number	Age	3.853	0.0064	**
5	F	w1118	Night Sleep Bout Number	Sex	18.92	<0.0001	****
6	A	CS	Day Sleep Bout Length	Interaction	2.516	0.0437	*
6	A	CS	Day Sleep Bout Length	Age	15.61	<0.0001	****
6	A	CS	Day Sleep Bout Length	Sex	14.02	<0.0001	****
6	B	Dah	Day Sleep Bout Length	Interaction	2.085	0.2174	ns
6	B	Dah	Day Sleep Bout Length	Age	5.208	0.0122	*
6	B	Dah	Day Sleep Bout Length	Sex	0.7957	0.1925	ns
6	C	w1118	Day Sleep Bout Length	Interaction	4.987	0.0052	**
6	C	w1118	Day Sleep Bout Length	Age	5.871	0.0019	**
6	C	w1118	Day Sleep Bout Length	Sex	3.189	0.0042	**
6	D	CS	Night Sleep Bout Length	Interaction	2.912	0.0059	**
6	D	CS	Night Sleep Bout Length	Age	38.07	<0.0001	****
6	D	CS	Night Sleep Bout Length	Sex	7.123	<0.0001	****
6	E	Dah	Night Sleep Bout Length	Interaction	4.646	0.0046	**
6	E	Dah	Night Sleep Bout Length	Age	6.601	0.0004	***
6	E	Dah	Night Sleep Bout Length	Sex	15.08	<0.0001	****
6	F	w1118	Night Sleep Bout Length	Interaction	0.4482	0.7304	ns
6	F	w1118	Night Sleep Bout Length	Age	4.122	0.0087	**
6	F	w1118	Night Sleep Bout Length	Sex	14.26	<0.0001	****
