# Supplementary material for: Sex- and strain-dependent effects of ageing on sleep and activity patterns in Drosophila
Source: PLoS One. 2024 Aug 16;19(8):e0308652. doi: 10.1371/journal.pone.0308652 (PMC11329114; doi:10.1371/journal.pone.0308652)
Supplement: S2 Fig — (A-F) Activity traces show the population mean +/- SEM of activity counts for each 30-minute bin across day and night cycles. (G-L) Box-and-whisker plots (minimum, 25%, median, 75%, maximum) show the summed activity counts during the day and night cycles for flies of each strain, sex, and age. n = 41–64 flies per condition; * p<0.05, ** p<0.01, **** p<0.0001 by Bonferroni multiple comparisons test. (PDF) [file pone.0308652.s002.pdf]

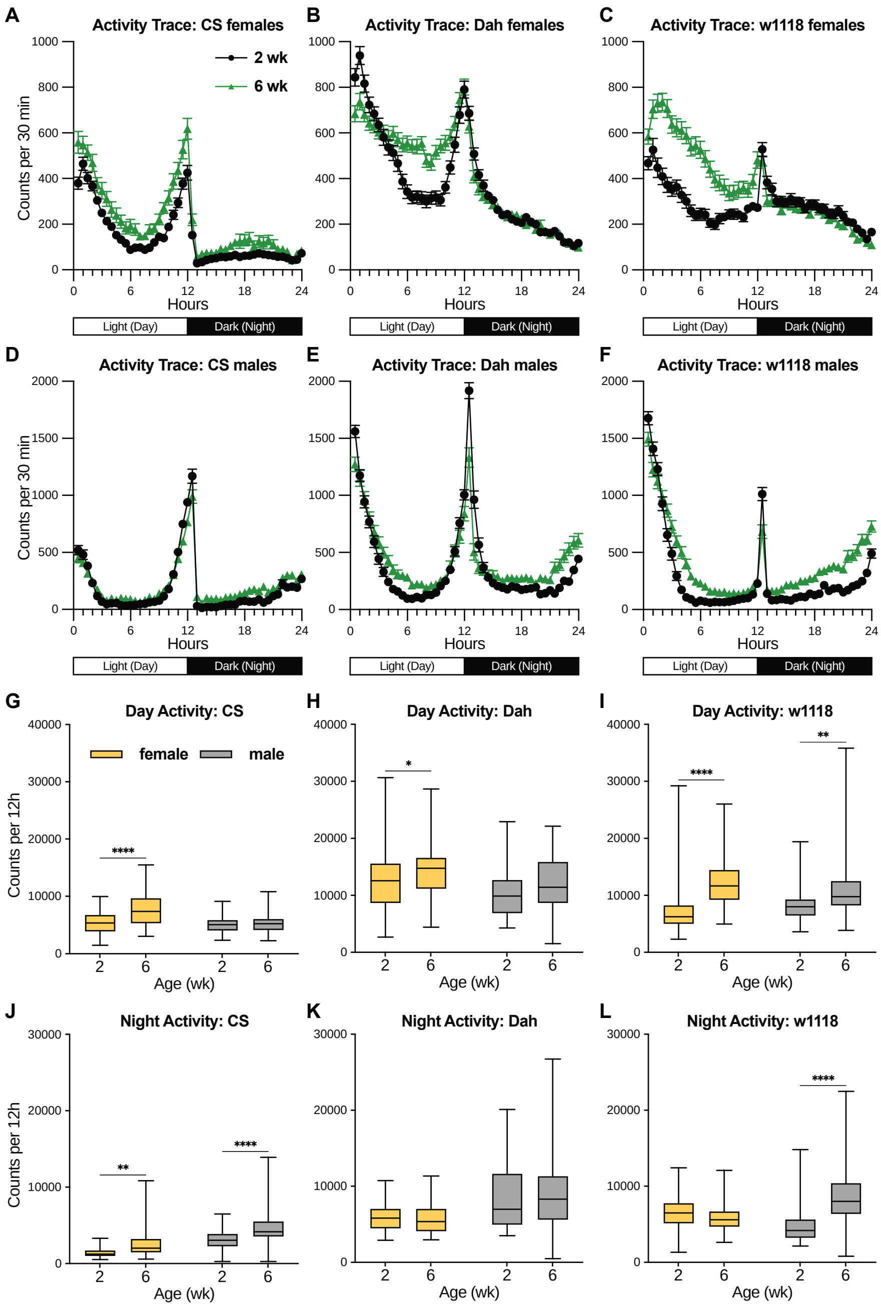

S2 Fig. Activity profiles of 2- and 6-week-old *CS*, *Dah*, and w^{1118} female and male flies, replicate experiments. (A-F) Activity traces show the population mean +/- SEM of activity counts for each 30-minute bin across day and night cycles. (G-L) Box-and-whisker plots (minimum, 25%, median, 75%, maximum) show the summed activity counts during the day and night cycles for flies of each strain, sex, and age. n=41-64 flies per condition; * p<0.05, ** p<0.01, **** p<0.0001 by Bonferroni multiple comparisons test.
