# Supplementary material for: Sex- and strain-dependent effects of ageing on sleep and activity patterns in Drosophila
Source: PLoS One. 2024 Aug 16;19(8):e0308652. doi: 10.1371/journal.pone.0308652 (PMC11329114; doi:10.1371/journal.pone.0308652)
Supplement: S5 Fig — (A-F) Box-and-whisker plots (minimum, 25%, median, 75%, maximum) show the number of independent sleep bouts (defined as 5 or more consecutive minutes of 0 activity counts) during the day and night cycles for flies of each strain, sex, and age. n = 41–64 flies per condition; ** p<0.01, *** p <0.001, **** p<0.0001 by Bonferroni multiple comparisons test. (PDF) [file pone.0308652.s005.pdf]

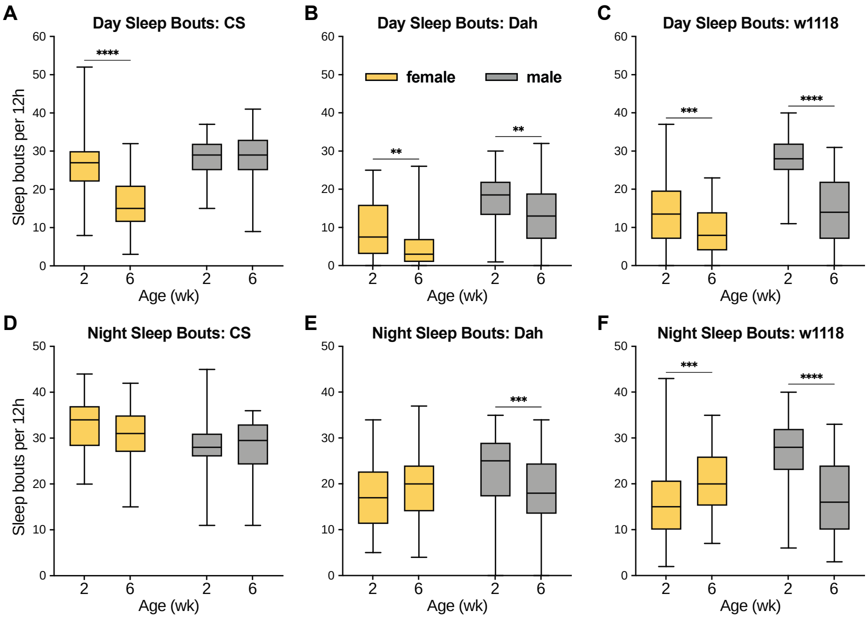


**S5 Fig. Sleep bout numbers in 2- and 6-week-old *CS*, *Dah*, and $w^{1118}$ female and male flies, replicate experiments**. (A-F) Box-and-whisker plots (minimum, 25%, median, 75%, maximum) show the number of independent sleep bouts (defined as 5 or more consecutive minutes of 0 activity counts) during the day and night cycles for flies of each strain, sex, and age. n=41-64 flies per condition; ** p<0.01, *** p <0.001, **** p<0.0001 by Bonferroni multiple comparisons test.
